# Bibliometric and visual analysis of gut microbiota research in functional bowel disorders from 2016 to 2025

**DOI:** 10.3389/fmed.2026.1735121

**Published:** 2026-01-28

**Authors:** Yujie Su, Xin Su, Zhengtao Chen, Lexun Wang, Jia Chen

**Affiliations:** 1 School of Chinese Medicine, Guangdong Pharmaceutical University, Guangzhou, China; 2 Institute of Chinese Medicine (Guangdong Metabolic Diseases Research Center of Integrated Chinese and Western Medicine), Guangdong Pharmaceutical University, Guangzhou, China

**Keywords:** bibliometric, functional bowel disorders, gut microbiota, gut-brain axis, hotspots, trends

## Abstract

**Objective:**

Research into Functional bowel disorders (FBDs) is increasingly focused on the role of gut microbiota (GM) in their pathogenesis and treatment. Nevertheless, a thorough and organized assessment of the existing research landscape remains absent. This study aimed to assess the research landscape, hotspots, and clinical advancements concerning GM in FBDs from 2016 to 2025, thereby providing a theoretical reference for future investigations.

**Methods:**

Publications from 2016 to 2025 were sourced from the Web of Science Core Collection and Scopus databases. These datasets were analyzed through a comprehensive bibliometric approach using R software, VOSviewer, and CiteSpace, with the resulting data visually represented for clearer interpretation. In addition, we collected clinical trials from PubMed during this period to evaluate advancements in the field.

**Results:**

From 2016 to 2025, the field of GM in FBDs exhibited a substantial overall increase in annual publications, with China being the most prolific contributor, followed by the United States, Italy, the United Kingdom, and Australia. The United States maintains the most extensive international collaboration network. At the institutional level, Mayo Clinic in the United States and University College Cork in Ireland emerged as the most active hubs for cooperative research. The journal *Nutrients* published the highest number of articles, while *Gastroenterology* garnered the greatest citation count. High-frequency keywords encompassed themes such as probiotics, double-blind, short-chain fatty acids, inflammation, and gut-brain axis. Current research emphasizes: (1) the mechanism by which GM influences FBDs via the gut-brain axis, (2) variations in the composition and metabolites of GM among different subtypes of FBDs, and (3) intervention strategies for treating FBDs through the modulation of GM. Clinical trials in this field have primarily concentrated on the role of core metabolites of the GM in symptom regulation in FBDs, the clinical application of integrated GM-modulating strategies, and the impact of specific GM abnormalities together with precision interventions.

**Conclusion:**

This study employed bibliometric and visual analytic approaches to provide a systematic overview of the research landscape and to identify key hotspots in GM research related to FBDs, offering critical insights that may guide future scientific investigations and clinical applications.

## 1 Introduction

Functional bowel disorders (FBDs) constitute a subgroup of disorders of gut-brain interaction characterized by chronic gastrointestinal symptoms primarily involving the mid-to-lower digestive tract (1). The core clinical manifestations include abdominal pain, bloating, and altered bowel habits. A fundamental hallmark of FBDs is the absence of definitive structural or biochemical abnormalities, rendering their diagnosis primarily reliant on established clinical criteria (2). According to the Rome IV criteria, FBDs are categorized into six subtypes: irritable bowel syndrome (IBS), functional constipation (FC), functional diarrhea, functional abdominal bloating/distension, unspecified FBD, and opioid-induced constipation (3). Epidemiological evidence shows that FBDs, which afflict an estimated 15–35% of people worldwide, significantly impair patients’ quality of life and generate substantial socioeconomic costs (4, 5).

A range of factors contribute to the development of FBDs, with psychological, physiological, and dietary components being particularly influential (1). The complex interplay among these elements is believed to be central to both the emergence and persistence of symptoms, ultimately shaping the course of the disorder. Evidence suggests that immune dysregulation, impaired intestinal barrier function, visceral hypersensitivity, and abnormal intestinal motility are central to disease manifestation (6). Psychological stress and comorbid emotional disturbances can further exacerbate symptoms (7). Dietary components, especially fermentable oligosaccharides, disaccharides, monosaccharides, and polyols, exert notable effects on intestinal motility, gas formation, and visceral hypersensitivity (8). Current clinical management strategies focus primarily on symptom control through dietary modification, pharmacologic therapy, and psychological intervention (9). However, the overall therapeutic effect varies, and the symptom relief for some patients remains unsatisfactory. Therefore, exploring new diagnostic and therapeutic strategies is a key focus of current clinical work.

Accumulating evidence over the past few years has emphasized the essential role of the GM in both the onset and pathophysiological processes of FBDs (10, 11). The GM performs essential physiological functions, including the synthesis of metabolites (12), maintenance of intestinal barrier integrity (13), regulation of host immunity (14), and modulation of neurodevelopment (15), among others. Balanced GM is essential for maintaining human health, while dysbiosis has been associated with a wide range of diseases, including inflammatory bowel disease, colorectal cancer, allergies, depression, diabetes, and obesity (16). Increasing evidence also implicates GM dysbiosis in FBDs (17, 18). Clinical studies have shown that individuals with IBS display significant alterations in GM composition compared to healthy controls, notably a decrease in the abundance of *Lactobacillus* (19). The fecal metabolite profiles of patients with FBDs, including short-chain fatty acids (SCFAs) and bile acids, exhibit marked differences when compared to those of healthy individuals (20). Furthermore, compositional alterations in GM are also associated with central psychological disturbances and peripheral intestinal symptoms, emphasizing its pivotal role in gut-brain communication (21, 22). Interventions targeting GM, such as probiotic supplementation, have demonstrated efficacy in alleviating FBD symptoms. When administered in adequate amounts, probiotics colonize the gastrointestinal tract, inhibit pathogenic microorganisms, and restore microbial equilibrium (23). Notably, despite the expanding body of research on GM in FBDs, analyses of research hotspots and emerging frontiers remain scarce. This gap impedes scholars from efficiently identifying emerging trends and future directions in the field.

Bibliometrics is a quantitative approach to analyzing published literature. It offers a powerful means to visualize knowledge structures, assess research trends, and identify influential contributors and topics (24). Compared to traditional narrative reviews, bibliometric analyses provide objective, quantitative insights and facilitate cross-comparisons across countries, institutions, and journals. Considering the rapid growth of research in this domain, we employed R software, VOSviewer, and CiteSpace for bibliometric and visual analysis of publications on GM in FBDs from 2016 to 2025. Such an approach delineates the current research landscape, uncovers knowledge gaps, and serves as a valuable reference for future experimental and clinical investigations. Ultimately, this work aims to offer comprehensive insights and practical references for advancing research and clinical practice related to GM in FBDs. Notably, although similar studies have been reported, our work represents the first comprehensive investigation specifically focusing on the GM in FBDS. We further compared our study with these similar investigations to delineate differences (Table 1). The overarching aim of this study is to facilitate a rapid and systematic understanding of this research field for investigators.

**Table 1:** Comparative analysis of bibliometric research.

Title	Research scope	Data sources	Time frame	Research hotspots and findings
Bibliometric and Visual Analysis of Gut Microbiota Research in Functional Bowel Disorders from 2016 to 2025	Disease spectrum: FBDs	WoSCC Scopus PubMed	2016–2025	1. Mechanism Networks: Elucidating the neuro-endocrine-immune integrative mechanisms of GM action via the gut-brain axis. 2. GM Similarities and Differences in FBD Subtypes: Systematic comparison of specificity and commonality in GM composition and metabolite profiles among different FBD subtypes. 3. Intervention Strategy Evaluation: Systematically reviewed evidence for various GM-targeted interventions (dietary modification, probiotics/prebiotics, microbiota transplantation).
Global research trajectories in gut microbiota and functional constipation: a bibliometric and visualization study	Single disease: FC	WoSCC	2013–2024	1. Core Mechanisms: GM influences gastrointestinal motility via the enteric nervous system; dysbiosis can trigger low-grade inflammation affecting intestinal function. 2. Intervention Studies: Probiotics and dietary fiber shown to improve bowel frequency and gut transit time.
A bibliometric analysis of global research status and trends in irritable bowel syndrome and gut microbiota metabolites	Single disease: IBS	WoSCC	1993–2024	1. Core Mechanisms: Microbial metabolites regulate the gut–brain axis to influence neural function and modulate inflammatory responses. 2. IBS Subtype-Specific Research: Currently, abnormal bile acid metabolism is primarily associated with IBS-D.
Research Status and Trends of Gut Microbiota and Intestinal Diseases Based on Bibliometrics	Disease spectrum: Intestinal Diseases	WoSCC	2009–2023	Research focuses on associations between specific diseases and microbiota: 1. IBS: *Bacillota*, *Bacteroidota*, etc. 2. Inflammatory bowel disease: *Faecalibacterium prausnitzii*, *Escherichia coli*, etc. 3. Colorectal Cancer: *Fusobacterium nucleatum*, *Morganella morganii*, etc. 4. Antibiotic-Associated Diarrhea: *Enterococcus*, *Akkermansia*, etc.
Global trends in research on irritable bowel syndrome and the brain–gut axis: Bibliometrics and visualization analysis	Single disease: IBS	WoSCC	2012–2021	Research focuses on the pathophysiological mechanisms by which microbial metabolites influence IBS via neuro-endocrine-immune pathways.

## 2 Materials and methods

### 2.1 Literature sources and search strategy

A systematic search of the Web of Science Core Collection (WoSCC) and Scopus databases was performed on September 14, 2025, to collect publications related to GM research in FBDs.

The WoSCC search formula was: TS = ((gut OR intestine OR bowel OR gastrointestine OR colon OR intestinal OR colorectal OR gastrointestinal OR enteric) AND (microbiome* OR microbiota* OR microbe* OR bacteria* OR microflora OR flora)) AND TS = (“functional bowel disorder*” OR “functional bowel disease*” OR “irritable bowel syndrome*” OR “irritable colon syndrome*” OR IBS OR “functional constipat*” OR “chronic idiopathic constipat*” OR “functional diarrh*” OR “functional abdominal bloat*” OR “functional abdominal distent*” OR “unspecified functional bowel disorder*” OR “opioid-induced constipat*”) (Note: The detailed search formulas for the Scopus database are provided in Supplementary material 1).

**Figure 1:**
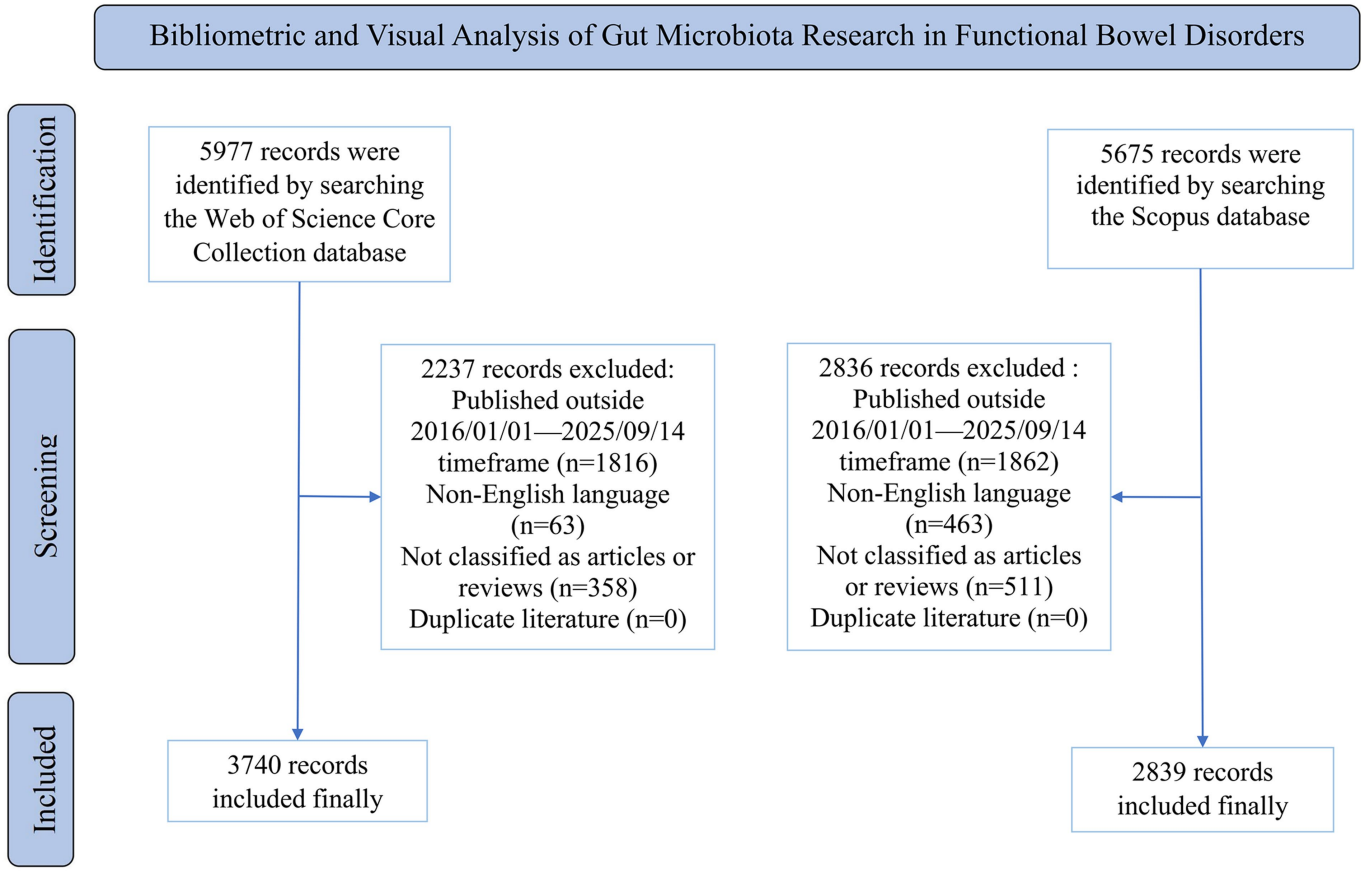
Flow chart of search strategy.

The search strategies are detailed in Figure 1. The literature retrieved from WoSCC was downloaded as full records with cited references and saved in plain text format. The literature obtained from Scopus was similarly downloaded as full records with cited references and saved in CSV format. In addition, relevant clinical trial data were retrieved from the PubMed database, and the detailed search strategy is provided in Supplementary material 1. Results of clinical trials were exported in PubMed format.

### 2.2 Data analysis

Given the differences in data formats between WoSCC and Scopus, merging datasets would result in data loss. Consequently, both databases were analyzed independently to preserve completeness and ensure reliability. Due to the superior data quality and citation precision of WoSCC, this dataset served as the primary focus of subsequent analyses, while results from Scopus were used for cross-validation.

Data visualization and bibliometric analyses were performed with the following software: (1) Annual publication trends were summarized using Microsoft Excel 2016. (2) Comprehensive bibliometric analysis was carried out in R software (version 4.5.1) employing the bibliometrix package (25). (3) Collaboration networks, co-citation maps, and keyword co-occurrence networks were constructed and visualized using VOSviewer (version 1.6.20) (26). (4) Citation burst detection was conducted with CiteSpace (version 6.4.1) (27). To enhance accuracy, two independent researchers conducted data extraction and verification.

The parameters in VOSviewer were standardized as follows: (1) In co-authorship network analysis, a minimum of ≥20 publications was required for countries and ≥15 for institutions. (2) In source co-citation analysis, each journal needed to have at least ≥500 citations. (3) In keyword co-occurrence analysis, the minimum keyword frequency was set at ≥30 for the WoSCC database and ≥80 for the Scopus database. To enhance analytical specificity, generic terms such as “gut microbiota” and “functional bowel disorders,” along with their synonyms, were excluded from the dataset. Information on journal impact factor (IF) was retrieved from the 2024 Journal Citation Reports (JCR).

## 3 Results

### 3.1 General landscapes of selected studies on GM in FBDs

**Figure 2:**
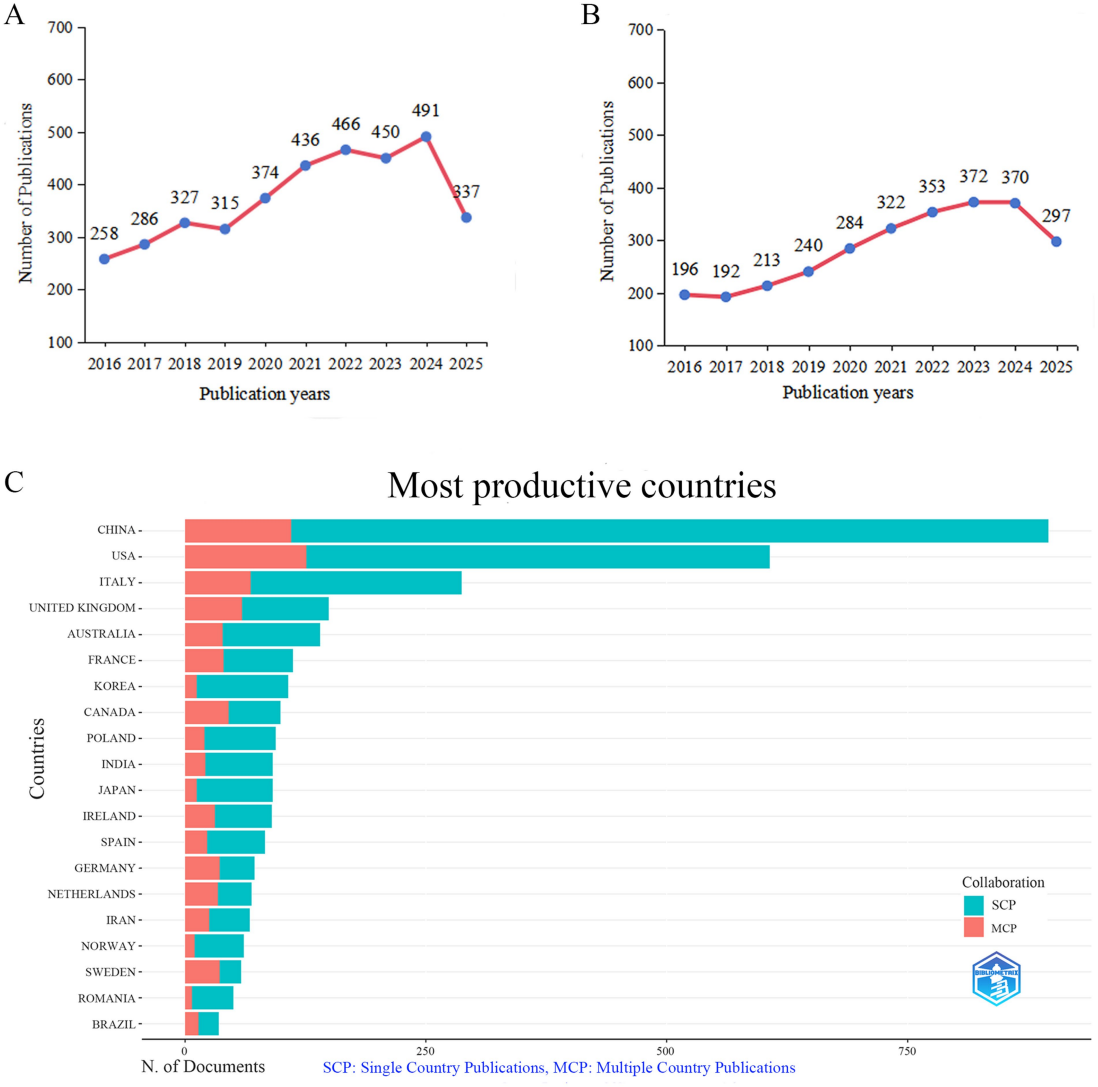
Annual publication trends in the field of GM in FBDs from 2016 to 2025. **(A,B)** Trends in publication results by year. **(C)** Country and collaboration distribution of corresponding authors. **(A,C)** Data sourced from WoSCC. **(B)** Data sourced from Scopus.

In total, 3,740 publications concerning GM in FBDs were identified from WoSCC. As illustrated in Figure 2A, annual publications show an overall upward trajectory over the past decade. The annual publication output demonstrated distinct phases: a period of gradual growth from 2016 to 2019 was followed by a rapid surge from 2019 to 2022. Subsequently, the number of publications stabilized at a high level with some fluctuation from 2022 to 2024. As of the search date (September 14, 2025), 337 articles had already been published within the year, further driving the growth of literature in this field. Similarly, 2,839 records were identified from Scopus, showing a broadly similar publication trend (Figure 2B). The annual publication trends in both databases reflect the expanding global attention to the role of GM in FBDs.

**Figure 3:**
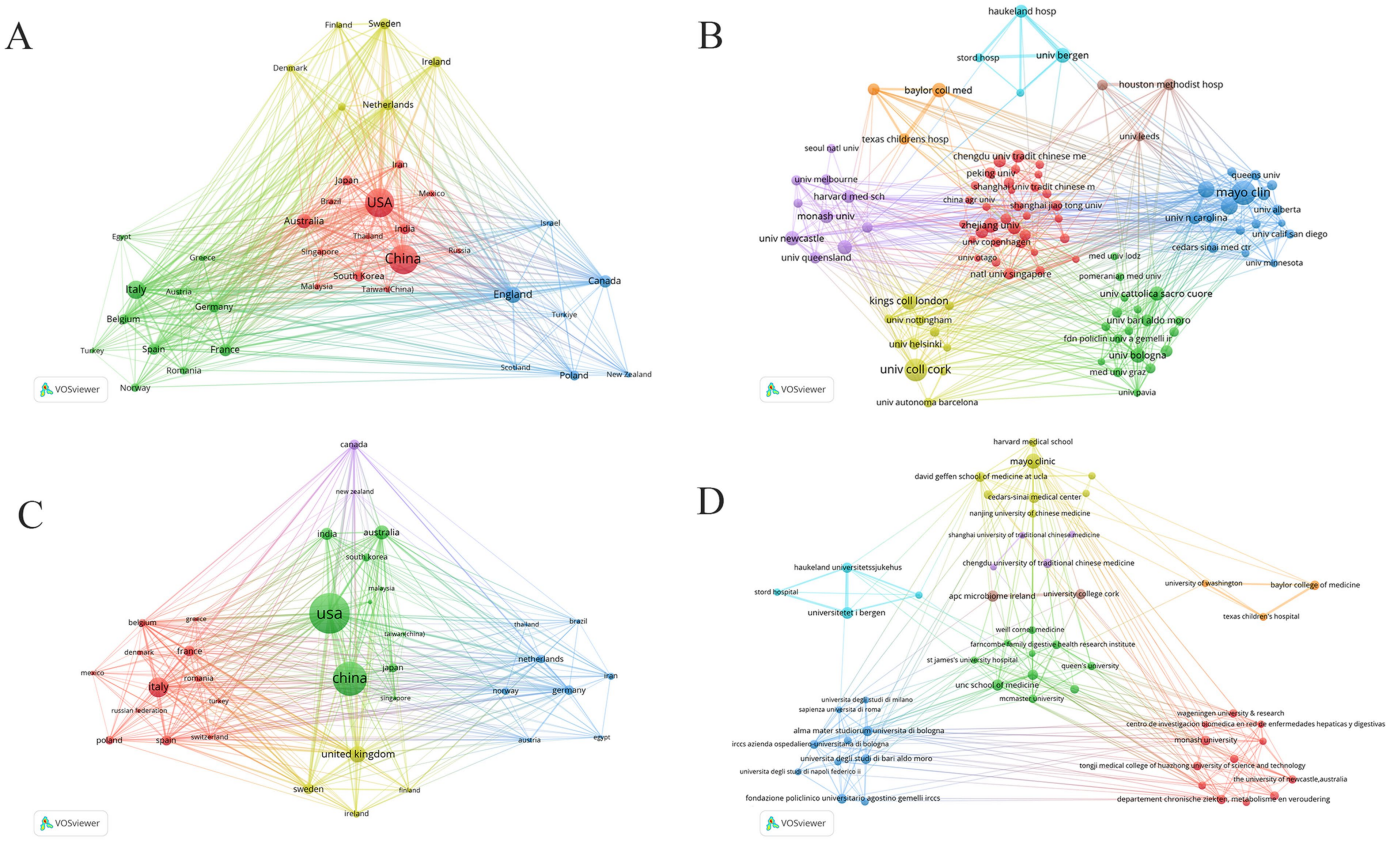
Collaboration network in the field of GM in FBDs from 2016 to 2025. **(A,C)** Cooperation between different countries. **(B,D)** Cooperation between different institutions. **(A,B)** Data sourced from WoSCC. **(C,D)** Data sourced from Scopus.

The distribution of corresponding authors’ countries revealed that China (*n* = 896) led in publications, followed by the United States (*n* = 607), Italy (*n* = 287), the United Kingdom (*n* = 149), and Australia (*n* = 140). Despite its dominant publication volume, China exhibited relatively limited international collaboration (Figure 2C; Table 2). In contrast, the United States exhibited the most extensive international collaboration network, linking closely with European and Asia-Pacific countries (Figure 3A). Institutional collaboration analysis (Figure 3B; Table 3) identified the Mayo Clinic (*n* = 102) and University College Cork (*n* = 91) as the primary research centers and hubs of international cooperation. The results from Scopus supported these findings, showing consistent patterns in inter-country and inter-institutional collaboration (Figures 3C,D).

**Table 2:** Most relevant countries by corresponding authors.

Country	Articles	SCP	Freq	MCP	MCP_Ratio
China	896	786	24	110	12.3
USA	607	481	16.2	126	20.8
Italy	287	219	7.7	68	23.7
United Kingdom	149	90	4	59	39.6
Australia	140	101	3.7	39	27.9
France	112	72	3	40	35.7
Korea	107	95	2.9	12	11.2
Canada	99	54	2.6	45	45.5
Poland	94	74	2.5	20	21.3
India	91	70	2.4	21	23.1
Japan	91	79	2.4	12	13.2
Ireland	90	59	2.4	31	34.4
Spain	83	60	2.2	23	27.7
Germany	72	36	1.9	36	50
Netherlands	69	35	1.8	34	49.3
Iran	67	42	1.8	25	37.3
Norway	61	51	1.6	10	16.4
Sweden	58	22	1.6	36	62.1
Romania	50	43	1.3	7	14
Brazil	35	21	0.9	14	40

MCP, multiple country publication; SCP, single country publication.

**Table 3:** Most relevant affiliations of the relationship.

Affiliation	Articles	Country
Mayo Clinic	102	USA
University College Cork	91	Ireland
McMaster University	56	Canada
University of Gothenburg	53	Sweden
King’s College London	50	United Kingdom
University of Bergen	46	Norway
Università di Bologna	44	Italy
Zhejiang University	44	China
Baylor College of Medicine	42	USA
University of Newcastle	42	Australia
University of California, Los Angeles	41	USA
Università Cattolica del Sacro Cuore	41	Italy
Monash University	39	Australia
Haukeland University Hospital	36	Norway
Jiangnan University	36	China
Harvard Medical School	35	USA
The University of North Carolina System	35	USA
Mayo Clinic	34	China
University College Cork	34	China
McMaster University	32	China

### 3.2 Analytical and visualization mapping of journals

To determine the most influential journals in research on GM in FBDs, publication and citation metrics were analyzed using the bibliometrix package in R software. The results were visualized with the ggplot2 package.

**Figure 4:**
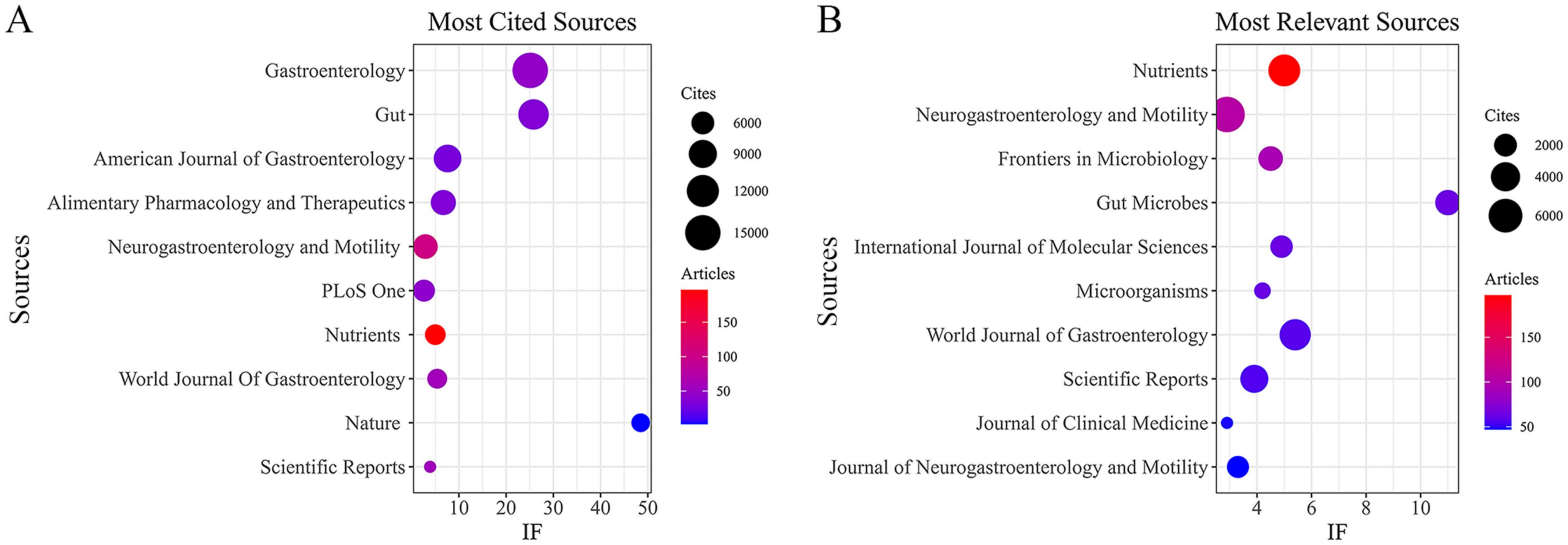
Journals with the most published and journals with the most cited. **(A)** Journals with the most published. **(B)** Journals with the most cited. **(A,B)** Data sourced from WoSCC.

**Figure 5:**
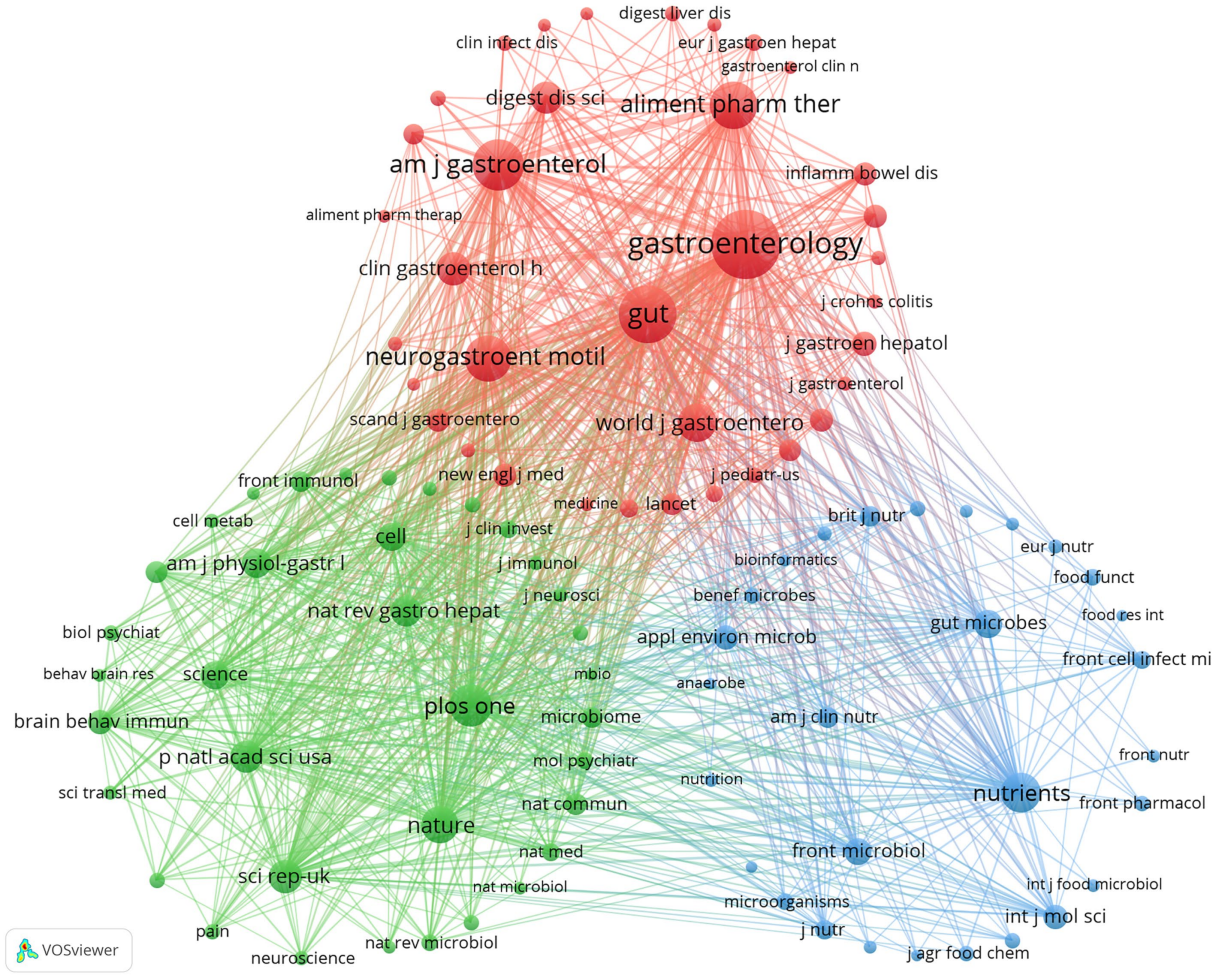
Co-cited journals involved in the field of GM in FBDs. Data sourced from WoSCC.

A total of 3,740 articles from 886 journals were identified from the WoSCC dataset (see Supplementary material 2 for detailed information). In terms of publication output (Table 4; Figure 4A), *Nutrients* (*n* = 197, IF = 5.0) had the highest number of publications, followed by *Neurogastroenterology and Motility* (*n* = 104, IF = 2.9), *Frontiers in Microbiology* (*n* = 94, IF = 4.5), *Gut Microbes* (*n* = 64, IF = 11.0), and *International Journal of Molecular Sciences* (*n* = 64, IF = 4.9). In terms of citation frequency (Table 5; Figure 4B), *Gastroenterology* ranked first (*n* = 15,143, IF = 25.1), followed by *Gut* (*n* = 10,657, IF = 25.8), *American Journal of Gastroenterology* (*n* = 8,676, IF = 7.6), *Alimentary Pharmacology and Therapeutics* (*n* = 7,166, IF = 6.7), and *Neurogastroenterology and Motility* (*n* = 6,864, IF = 2.9). As shown in Figure 5, the co-citation analysis indicated that *Gastroenterology*, *Gut*, and *American Journal of Gastroenterology* are central collaborative hubs in this field, exerting substantial influence. These results indicate that journals with higher impact factors generally publish a smaller number of studies in this domain, underscoring the necessity to strengthen the methodological depth, analytical rigor, and overall quality of research in this area. We also identified 2,839 articles from 883 academic journals from the Scopus dataset (see Supplementary material 3 for detailed information). The journals with higher publication volumes largely align with the findings from the analysis of data sourced from WOSCC.

**Table 4:** Top 10 journals with the most published.

Sources	Articles	IF (2024)	Cites
Nutrients	197	5	5,116
Neurogastroenterology and Motility	104	2.9	6,864
Frontiers in Microbiology	94	4.5	2,460
Gut Microbes	64	11	2,669
International Journal of Molecular Sciences	64	4.9	1914
Microorganisms	63	4.2	932
World Journal of Gastroenterology	58	5.4	4,823
Scientific Reports	56	3.9	3,550
Journal of Clinical Medicine	48	2.9	707
Journal of Neurogastroenterology and Motility	47	3.3	1833

IF, impact factor.

**Table 5:** Top 10 journals with the most cited.

Sources	Cites	IF (2024)	Articles
Gastroenterology	15,143	25.1	46
Gut	10,657	25.8	36
American Journal of Gastroenterology	8,676	7.6	30
Alimentary Pharmacology and Therapeutics	7,166	6.7	35
Neurogastroenterology and Motility	6,864	2.9	104
PLoS One	5,631	2.6	43
Nutrients	5,116	5	197
World Journal of Gastroenterology	4,823	5.4	58
Nature	4,475	48.5	2
Scientific Reports	3,550	3.9	56

IF, impact factor.

### 3.3 Citation bursts

**Figure 6:**
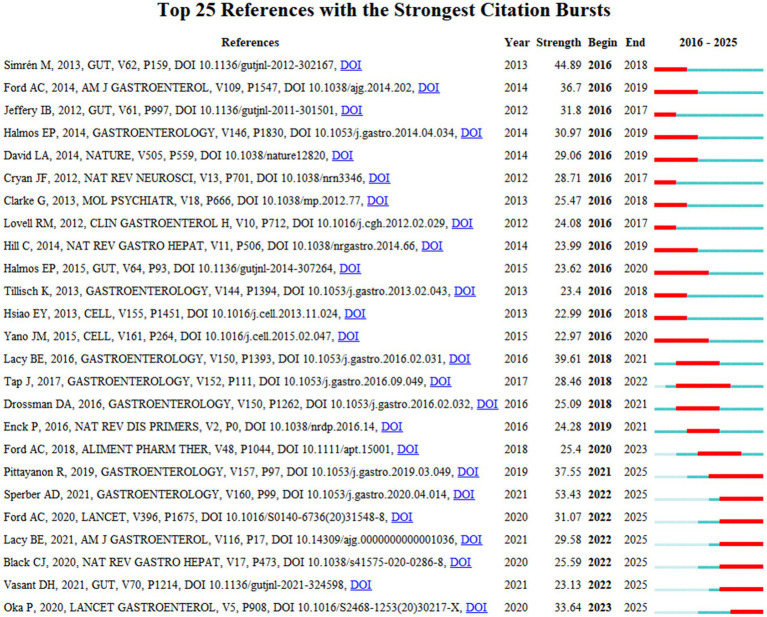
Top 25 references with the strongest citation bursts in the field of GM in FBDs. Data sourced from WoSCC.

To identify influential papers and frontier research trends, we employed CiteSpace to detect the 25 references exhibiting the most significant citation bursts from the WoSCC dataset (Figure 6). The titles and DOIs of these references are presented in Supplementary material 4.

Notably, the three references with the strongest citation bursts were: (1) “Worldwide Prevalence and Burden of Functional Gastrointestinal Disorders, Results of Rome Foundation Global Study (strength: 53.43);” (2) “Intestinal microbiota in functional bowel disorders: a Rome foundation report (strength: 44.89);” (3) “Bowel Disorders (strength: 39.61).” Furthermore, the three most recent bursts representing frontier research corresponded to the following publications: (1) “Global prevalence of irritable bowel syndrome according to Rome III or IV criteria: a systematic review and meta-analysis;” (2) “British Society of Gastroenterology guidelines on the management of irritable bowel syndrome;” (3) “Global burden of irritable bowel syndrome: trends, predictions and risk factors.”

The citation bursts analysis identified three primary research areas: (1) the mechanistic link of the gut-brain axis in FBD development; (2) distinct GM profiles associated with subtypes of IBS; and (3) the regulation of FBD symptoms by GM through dietary intervention.

### 3.4 Keyword clusters and thematic evolution

**Figure 7:**
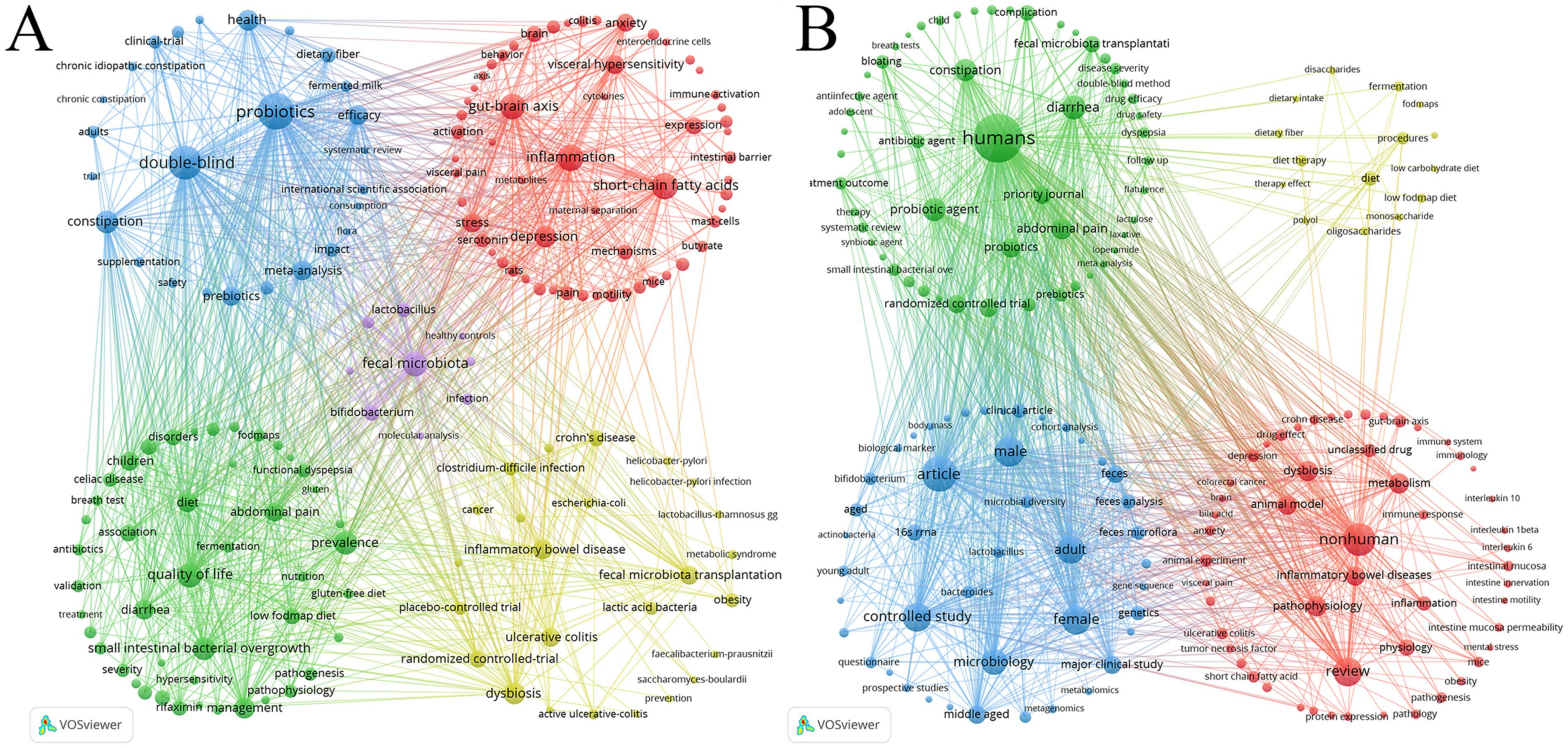
Keywords co-occurrence map of publications on the field of GM in FBDs. **(A)** Data sourced from WoSCC. **(B)** Data sourced from Scopus.

Keyword co-occurrence analysis reveals major research themes. In this study we identified 10,407 keywords from the WoSCC dataset using VOSviewer. Table 6 presents the top 20 high-frequency keywords with “probiotics” (*n* = 711) “double-blind” (*n* = 611) “short-chain fatty acids” (*n* = 358)“inflammation” (*n* = 351) “gut-brain axis” (*n* = 336) and “quality of life” (*n* = 321) ranking at the forefront. Further analysis of keywords with an occurrence frequency of ≥30 resulted in 165 keywords from which a keyword clustering map was generated (Figure 7A). The clustering analysis revealed five major research hotspots as follows: (1) The neuroimmune mechanism of GM regulation of FBDs via the gut-brain axis (red dots) including 59 keywords. In this cluster “gut-brain axis” serves as the central concept with many related keywords such as “short-chain fatty acids,” “butyrate,” and “serotonin” that are key signaling molecules in this axis. Play a crucial role in the communication between the gut and the brain. Keywords like “immune activation,” “cytokines,” and “oxidative stress” suggest that changes in the GM may lead to immune dysregulation. The inclusion of “depression,” “visceral hypersensitivity,” and “intestinal barrier” highlights the bidirectional regulation between the gut and the brain as well as the neuroimmune mechanisms involved. (2) Dietary interventions on GM metabolic activity and symptom management in FBDs (green dots) including 46 keywords. This cluster includes keywords such as “low-FODMAP diet” and “gluten-free diet,” which represent specific dietary interventions aimed at managing FBDs. Keywords like “fermentation,” “hydrogen,” and “methane” highlight the role of dietary interventions in modulating the metabolic activity of the GM. The research also emphasizes factors like “quality of life,” “reduces symptoms,” and “nutrition,” underscoring the potential of diet to improve patient outcomes and alleviate symptoms. Collectively these keywords outline a research paradigm that links specific dietary inputs to microbial metabolic outputs ultimately aiming for symptom relief and improved quality of life in FBDs management. (3) Evidence-based investigations into the roles of probiotics and prebiotics in FBDs (blue dots) including 26 keywords. This cluster highlights keywords such as “probiotics,” “prebiotics,” and “health,” underscoring the role of probiotics and prebiotics in improving gut health in FBDs. Keywords like “efficacy,” “impact,” and “safety” indicate that the research focuses on evaluating the efficacy and safety of these interventions. Keywords such as “systematic review,” “meta-analysis,” and “double-blind” reflect that the research primarily centers on validating the application of probiotics and prebiotics in FBDs management through clinical trials and systematic reviews emphasizing their reliability as therapeutic tools. (4) The clinical application and therapeutic efficacy of probiotics and fecal microbiota transplantation (FMT) in FBDs management (yellow dots) including 25 keywords. This cluster includes keywords such as “fecal microbiota transplantation,” “*saccharomyces-boulardii*,” and “lactic acid bacteria,” which indicate the therapeutic potential of probiotics and FMT in managing FBDs. Keywords like “dysbiosis,” “ulcerative colitis,” and “antibiotic-associated diarrhea” highlight the connection between GM imbalance and gastrointestinal diseases. Keywords such as “randomized controlled-trial,” “placebo-controlled trial,” and “prevention” emphasize the clinical validation and effectiveness of these therapies. Overall, these keywords underscore the therapeutic potential of probiotics and FMT in restoring GM balance and improving FBDs symptoms. (5) Comparative analysis of GM diversity between healthy individuals and patients with FBDs (purple dots) including 9 keywords. This cluster includes keywords such as “healthy controls,” “molecular analysis,” and “identification,” highlighting the approach of comparative studies and molecular identification to reveal differences in the GM between healthy individuals and patients with FBDs. Keywords like “fecal microbiota,” “*Bifidobacterium*,” and “diversity” underscore the distinctions in the GM between healthy individuals and those with FBDs. These keywords suggest that analyzing microbiota diversity is a crucial tool for understanding the pathophysiological mechanisms of FBDs. All keywords corresponding to these five clusters are listed in Supplementary material 5.

**Table 6:** The top 20 keywords.

Rank	Keywords (WoSCC)	Count	Keywords (Scopus)	Count
1	probiotics	711	humans	2,303
2	double-blind	611	article	1,315
3	short-chain fatty acids	358	nonhuman	1,165
4	inflammation	351	male	1,020
5	gut-brain axis	336	female	950
6	quality of life	321	review	950
7	prevalence	298	controlled study	936
8	small intestinal bacterial overgrowth	277	adult	893
9	dysbiosis	256	microbiology	782
10	depression	243	diarrhea	722
11	health	229	probiotic agent	659
12	management	221	constipation	628
13	inflammatory bowel disease	215	abdominal pain	557
14	diet	211	pathophysiology	528
15	efficacy	205	metabolism	525
16	diarrhea	203	dysbiosis	514
17	meta-analysis	197	animal model	511
18	fecal microbiota transplantation	195	inflammatory bowel diseases	499
19	stress	192	probiotics	488
20	prebiotics	192	priority journal	457

Additionally, 16,619 keywords were identified from the Scopus dataset. Table 6 presents the top 20 high-frequency keywords with an occurrence frequency of ≥457. Further screening identified 168 keywords with an occurrence frequency of ≥80, and a keyword clustering map was drawn (Figure 7B). Currently, research on GM and FBDs focuses on four key hotspots: (1) the role of GM imbalance in the pathophysiology of FBDs (red dots); (2) the management of FBD symptoms to improve quality of life through GM intervention (green dots); (3) multi-omics Insights into GM characteristics in FBD Patients (blue dots); and (4) the impact of the low FODMAP diet on GM and FBD symptoms (yellow dots). All keywords corresponding to these four clusters are listed in Supplementary material 6.

**Figure 8:**
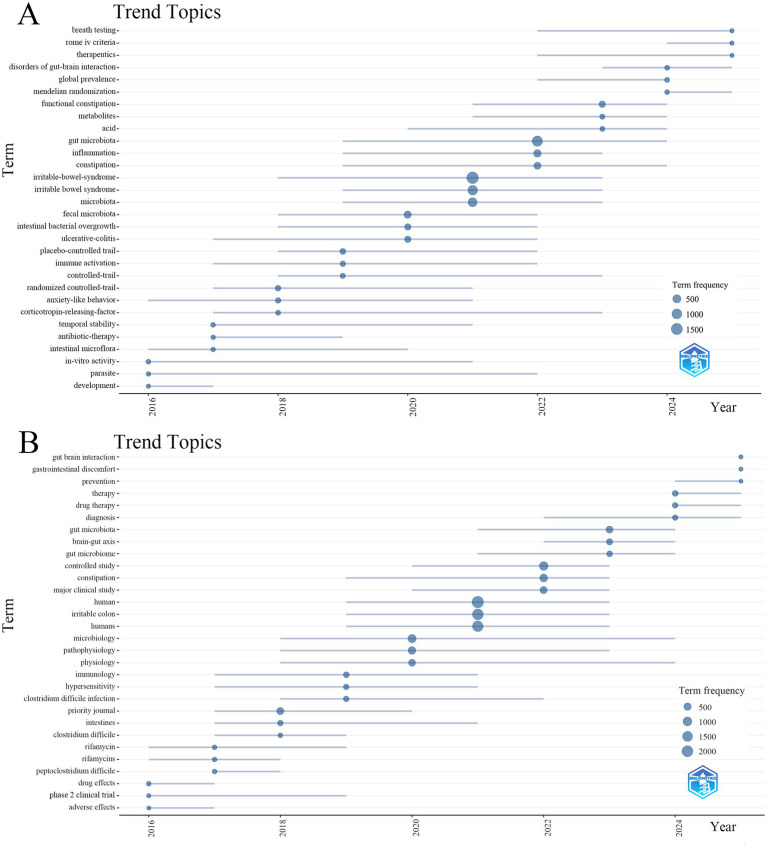
Trend topics in the field of GM in FBDs research. **(A)** Data sourced from WoSCC. **(B)** Data sourced from Scopus.

To identify evolving research trends and future directions, we employed the bibliometrix package in R software to analyze topical trends based on the WoSCC dataset (Figure 8A). The analysis reveals a clear thematic evolution over time. From 2016 to 2018, the field was primarily dedicated to the fundamental exploration of the gut-brain axis and the role of GM in the pathophysiology of FBDs, based primarily on basic and animal studies. The period from 2019 to 2021 was marked by advancements in research methods, particularly the increasing prominence of placebo-controlled trials and randomized controlled trials, which significantly enhanced the rigor and scientific quality of studies. Meanwhile, the connection between GM and the immune system emerged as a key research direction. Between 2022 and 2023, research extended to multi-omics and metabolomic analyses, highlighting microbial metabolism, intestinal function, and disease subtype differentiation. Since 2024, research has entered a stage of interdisciplinary integration and clinical standardization, emphasizing diagnostic criteria, bioinformatics, and precision-based microbiota interventions. Building on this foundation, future research is expected to place greater emphasis on early warning mechanisms for disease, effects of GM interventions across different populations, and more precise treatment strategies. Furthermore, the same topic trend analysis conducted on the Scopus dataset was broadly consistent with the WoSCC results (Figure 8B).

To further confirm these future research trends and highlight the latest advancements in the field, we reviewed 337 papers published in 2025 from the WoSCC dataset (see Supplementary material 7 for detailed information). The findings from this recent research confirm that GM studies related to FBDs are increasingly focusing on clinical applications and precision medicine. Key developments include the use of multi-omics techniques for advancing diagnostic biomarkers and an enhanced understanding of brain-gut interactions, particularly the neuroimmune mechanisms involved. Moreover, as the role of GM in specific FBD subtypes continues to attract attention, personalized interventions tailored to individual microbiota profiles are becoming more prominent.

Overall, the field has gradually shifted from early pathological and clinical observations to a balance between mechanism exploration and clinical application, with future research moving toward precision, evidence-based approaches, and interdisciplinary integration.

### 3.5 Comprehensive analysis of research hotspots

To gain a more holistic understanding of current focal points, we integrated results from citation bursts, keyword frequency, keyword clusters, and thematic evolution. The results demonstrate that the research hotspots in this area cluster around three principal directions, as follows: (1) the mechanisms by which GM influences FBDs via the gut-brain axis, involving a complex network encompassing the nervous, immune, and endocrine pathways; (2) variations in the composition and metabolites of GM among different subtypes of FBDs, which drive research into biomarkers for precise diagnosis and targeted therapy; and (3) intervention strategies for treating FBDs through the modulation of GM, particularly via specific dietary patterns, probiotics, prebiotics, and FMT.

### 3.6 Clinical progress analysis

A total of 57 clinical trials were retrieved from the PubMed database (see Supplementary material 8 for detailed information). These studies can be categorized into three major research themes: (1) the role of core metabolites of the GM in symptom regulation in FBDs; (2) the clinical application of integrated GM-modulating strategies in FBDs; and (3) the impact of specific GM abnormalities in FBDs and the application of precision interventions.

## 4 Discussion

### 4.1 General information

Between 2016 and 2025, the study identified 3,740 publications in WoSCC and 2,839 articles indexed in Scopus. As the general information obtained from the two databases was highly consistent, the analysis is presented based on the WoSCC dataset. From 2016 to 2025, publications addressing GM in FBDs exhibited an overall upward trend, underscoring the increasing attention devoted to GM’s role in FBDs.

Among the countries contributing to this field, China led in publication output, with substantial contributions also observed from the United States, Italy, the United Kingdom, and Australia. Notably, despite China’s leading output, the United States exhibited more extensive international collaboration. Within the top 20 publishing institutions, five were based in the United States and five in China, with the remaining institutions located in Australia and several European countries. The absence of a single dominant institution, together with this wide geographic distribution, reflects a highly competitive yet collaborative global research landscape. Encouraging broader international collaboration in future studies will be essential for improving the generalizability and overall impact of research findings.

Journal analysis showed that the 3,740 publications were distributed across 886 journals. Journals such as *Nutrients*, *Neurogastroenterology and Motility*, *Frontiers in Microbiology*, *Gut Microbes*, and *International Journal of Molecular Sciences* had higher publication volumes and made substantial contributions. High-impact journals such as *Gastroenterology* and *Gut* were highly cited but published fewer papers in this field. The results suggest that these journals are crucial platforms for advancing scientific knowledge on GM in FBDs.

### 4.2 Research hotspots and development trends

By integrating multiple analytical approaches, such as citation burst, keyword frequency, keyword clusters, and thematic evolution, this study elucidated the key research domains within GM research in FBDs. The findings indicate that current cutting-edge studies and newly emerging focal points in this field primarily concentrate on three main thematic domains. First, the mechanism by which GM influences FBDs via the gut-brain axis. Second, variations in the composition and metabolites of GM among different subtypes of FBDs. Finally, intervention strategies for treating FBDs through the modulation of GM.

#### 4.2.1 The mechanism by which GM influences FBDs via the gut-brain axis

Studies have revealed that the GM significantly contributes to the onset and development of FBDs by influencing the gut-brain axis (28). The gut–brain axis constitutes a two-way communication system linking the intestine and the central nervous system, regulating intricate interactions through neural, endocrine, and immune pathways mediated by various signaling molecules (29). GM can directly impact the central nervous system through microbial metabolites or indirectly influence brain function by stimulating enteric nerves, modulating neuroendocrine signaling, and eliciting immune responses (30). These pathways are closely linked to the pathophysiology of FBDs.

With advancements in multiple omics technologies, the regulatory role of GM metabolites in FBDs has been more clearly elucidated (31, 32). The GM modulates bidirectional communication between the intestine and the brain through the generation of various bioactive metabolites, such as SCFAs, secondary bile acids, and tryptophan-derived compounds (33). SCFAs, such as butyrate, propionate, and acetate, generated through microbial metabolism, are recognized as key signaling agents in the gut-brain axis (34). As small-molecule metabolites, SCFAs can engage with enteroendocrine cells to modulate hormonal signaling, traverse the intestinal epithelium into systemic circulation, and even penetrate the blood–brain barrier, thereby exerting effects on the central nervous system (35, 36). An experimental study has demonstrated that SCFAs can influence FBDs by acting on intestinal chromaffin cells to modulate serotonin synthesis and release (37). Given the pivotal role of serotonin in regulating both central and peripheral nervous system activities, this observation suggests a possible pathway through which the GM may influence FBDs via the gut-brain axis (38).

Beyond metabolites, GM dysbiosis can also impact the gut-brain axis to promote the pathophysiology of FBDs by activating the intestinal mucosal immune system and disrupting neuroendocrine homeostasis. A comparative study of IBS rat models provides compelling evidence for this integrated dysregulation. The study revealed that GM alterations were accompanied by significant increases in serum levels of serotonin, substance P, and corticotropin-releasing factor, along with a marked decrease in neuropeptide Y (39). For instance, under stress conditions, GM alterations may activate intestinal mucosal mast cells to release neurotransmitters and pro-inflammatory factors (40). This process will lead to visceral hypersensitivity and immune activation, which are hallmark features of FBDs. These immune-derived mediators can directly sensitize the terminals of visceral afferent nerves, lowering their threshold for activation and resulting in the perception of pain from normal bowel movements (41). Moreover, the gut-brain axis provides a valuable perspective for exploring the bidirectional interaction linking FBDs to psychological conditions (42). Alterations in gut microbial composition can contribute to anxiety and depression through neuroimmune and endocrine pathways (43). In turn, these negative emotional states can exacerbate FBDs by modulating gut physiology and the microbial ecosystem, thereby creating a vicious cycle (44, 45).

Overall, the mechanism through which GM regulates FBDs via the gut-brain axis remains to be fully elucidated. Future research should explore the relationship between gut-brain axis signaling molecules and GM function to provide a foundation for novel intervention strategies for FBDs.

#### 4.2.2 Variations in the composition and metabolites of GM among different subtypes of FBDs

Analysis of keywords and related information has revealed the relationship between GM characteristics and different subtypes of FBDs as a major research focus. GM dysbiosis is prevalent among patients with FBDs. Its characteristics do not stem from the infection of a single pathogen; instead, they involve changes in the structure, richness, and stability of the overall microbial ecosystem (46). Crucially, this dysbiosis is not uniform but manifests as highly specific compositional and metabolic signatures of the GM that correlate with distinct clinical symptoms.

In healthy individuals, the phyla *Bacteroidota* and *Bacillota* together make up nearly 90% of the total intestinal microbiota, while *Pseudomonadota*, *Actinobacteriota*, and *Verrucomicrobiota* represent smaller proportions (47). IBS is the most extensively studied FBD subtype. Research has demonstrated that the GM composition in individuals with IBS is altered, characterized by an increased *Bacillota*/*Bacteroidota* ratio and a higher abundance of *Actinobacteriota* and *Verrucomicrobiota* (48). The most notable fecal bacterial markers in IBS patients are *Clostridia* and *Veillonella* (49). Elevated levels of these bacteria are associated with diarrhea, whereas reduced levels are linked to constipation. In addition to differences in microbial composition, diarrhea-predominant IBS patients exhibit higher levels of bile acids, polyamines, and glycolysis intermediates (e.g., malic acid and fumaric acid) compared to constipation-predominant IBS (IBS-C) patients (50). These results suggest that microbial metabolic activity may directly influence defecation patterns.

Conversely, conditions characterized by constipation, such as IBS-C and FC, largely share similar microbial features with slight variations. Research indicates that the richness and diversity of GM in FC patients are significantly increased, with an enrichment of potentially pathogenic bacteria, including *Intestinibacter*, *Klebsiella*, and *Akkermansia* (51). In contrast, while no significant difference in overall GM diversity was observed between IBS-C patients and healthy individuals, specific alterations at the species level have been reported, including a significant reduction in beneficial bacteria like *Megasphaera elsdenii*, *Bifidobacterium bifidum*, and *Alistipes inops*, and an increased abundance of *Lactobacillus iners* (48). At the metabolic level, these conditions exhibit some common signatures. For instance, concentrations of SCFAs are significantly reduced in both IBS-C and FC, thereby diminishing their stimulatory effects on enteric nerves and muscles and contributing to delayed colonic transit (20, 48). Similarly, the concentrations of deoxycholic acid, which exerts pro-motility effects, and its precursor chenodeoxycholic acid are also decreased (20, 52). Furthermore, in IBS-C, pro-inflammatory metabolites such as leukotriene D5 are elevated, which is associated with intestinal barrier damage and low-grade inflammation and likely underlies the abdominal pain characteristic of this disorder (48).

Metagenomics and metabolomics are currently cornerstone methodologies for studying GM, enabling comprehensive analysis of microbial composition and metabolites. Metagenomics enables the identification of characteristic microbial taxa and gene functions correlated with different subtypes of FBDs (53, 54). Metabolomics, in contrast, provides direct insights into the terminal products of microbial metabolic activity (55, 56), including specific SCFAs, bile acid profiles, intestinal gases, and inflammation-related lipids (57). By integrating and correlating metagenomic and metabolomic datasets, a multidimensional biomarker framework can be developed to inform both the diagnosis and therapeutic decision-making of FBDs.

By analyzing the composition and metabolites of GM, we aim to address the subjectivity inherent in the current diagnosis of FBDs and facilitate a shift from symptom-based descriptions to classifications grounded in biological mechanisms. Future research is warranted to validate the sensitivity and specificity of these biomarkers in large-scale prospective cohorts and to advance their translation into standardized clinical diagnostic tools. Concurrently, the potential of these markers as therapeutic targets should be investigated to inform the personalized treatment of FBDs.

#### 4.2.3 Intervention strategies for treating FBDs through the modulation of GM

Our analysis reveals that strategies targeting the GM have emerged as a major research focus in the treatment of FBDs. GM modulation for FBDs may be accomplished through a range of strategies, including dietary interventions, probiotics, prebiotics, and FMT.

The low FODMAP diet (LFD) is currently the most widely recommended dietary intervention for FBDs and has shown a significant influence (58). FODMAPs, which stand for fermentable oligosaccharides, disaccharides, monosaccharides, and polyols, are short-chain carbohydrates and sugar alcohols that are inadequately absorbed in the small intestine and undergo rapid fermentation by GM (59). Research has demonstrated that a high FODMAP diet significantly shortens colonic transit time and reduces intestinal bacterial diversity (60). However, the implementation of LFD in clinical practice is complex. If patients follow the LFD without professional guidance, it may lead to issues such as difficulties in self-management and restricted social activities (61). Due to challenges in implementing the LFD, the Mediterranean diet, which is easier to adhere to and can maintain beneficial regulation of the intestinal microbiota, may serve as an alternative (62).

Alongside dietary interventions, both probiotics and prebiotics are crucial in the management of FBDs. Probiotics consist of live microorganisms that, when consumed in sufficient quantities, exert positive influences on the health of the host (63). Their beneficial effects are primarily mediated through modulation of the GM, including alterations in microbial composition, metabolic activity, and host immune responses (64). For example, specific *Lactobacillus plantarum* strains effectively rebalance the GM in individuals with functional diarrhea and diarrhea-predominant IBS by reducing populations of potentially detrimental bacteria such as *Bacteroides* and *Eggerthella* and by enriching beneficial genera including *Akkermansia* and *Anaerostipes*, leading to improved bowel habits (65). In contrast, prebiotics are non-digestible dietary substrates that specifically enhance the growth and metabolic activity of beneficial bacteria, contributing to enhanced stability and functional resilience of the intestinal ecosystem (63). Common prebiotics, such as fructooligosaccharides and inulin, can indirectly regulate GM structure by providing a growth environment for beneficial bacteria and improving intestinal function (66, 67). A study has demonstrated that synbiotics, composed of probiotics and prebiotics, can enhance intestinal barrier function, inhibit intestinal inflammation, and effectively alleviate IBS and related colonic dysfunction by modulating the intestinal microbiota (68).

Beyond these targeted interventions, FMT represents a more comprehensive strategy for modulating the GM. FMT involves the transfer of processed fecal material from a healthy donor into the recipient’s gastrointestinal tract to reintroduce a diverse microbial community (69). The primary goal of this procedure is to suppress pathogenic bacteria, restore microbial balance, and achieve therapeutic benefits (70). Evidence from clinical research has shown that a three-month FMT regimen markedly boosts the prevalence of beneficial bacterial taxa such as *Akkermansia* and *Prevotella*, thereby effectively restoring microbial diversity and promoting a more stable and functionally resilient gut ecosystem (71). Moreover, emerging evidence indicates that FMT plays a modulatory role in both diarrhea-predominant and constipation-predominant disorders (72, 73). Notably, following antibiotic-induced disruption of the human GM, autologous fecal transplantation has been shown to repair intestinal mucosal integrity and reestablish structural homeostasis, ultimately restoring microbial equilibrium within the host (74).

Looking ahead, integrated intervention strategies are likely to emerge, combining pharmacological treatments, dietary interventions, probiotics, prebiotics, and FMT to provide more precise and personalized treatment plans for patients. This interdisciplinary and individualized treatment model is expected to become the future standard for managing FBDs.

### 4.3 Clinical progress

This study comprehensively evaluated 57 clinical trials and clarified the key trends and key areas of focus in current clinical research on the GM in FBDs: (1) Symptom regulation by SCFAs in FBDs. Clinical evidence indicates that levels of SCFAs in patients with FBDs, particularly butyrate levels, are closely associated with the severity of gastrointestinal symptoms (75). For example, increased butyrate levels are significantly correlated with symptom improvement in patients with FBDs, suggesting that butyrate may exert therapeutic effects by enhancing epithelial barrier integrity and modulating local immune responses (76). Further studies have demonstrated that enrichment of butyrate-producing bacteria effectively alleviates abdominal pain in patients with IBS-D (77). (2) Probiotic-centered combination therapy as a strategy for modulating the GM in FBDs. Probiotics used in combination with other therapeutic approaches or as multi-strain formulations can exert synergistic effects and provide more sustained clinical benefits than single-strain probiotics. For instance, probiotic supplementation has been shown to mitigate the reduction in *Bifidobacterium* induced by a LFD, thereby helping maintain microbial diversity (78). In addition, multi-strain formulations have been demonstrated to alter GM composition and improve clinical symptoms (79, 80). A compound preparation containing *Lactobacillus*, *Bifidobacterium*, xylo-oligosaccharides, and dietary fiber has been reported to significantly improve defecation perception in patients with FC, accompanied by a decreased proportion of *Bacillota* and an increased proportion of *Bacteroidota* in the GM (78). Collectively, these studies indicate that probiotics and their combined interventions have broad clinical application potential in FBDs. (3) Impact of specific GM abnormalities in FBDs and microbiota-targeted therapy. Evidence suggests that a Clostridium-enriched GM can induce diarrhea in patients with IBS-D by disrupting bile acid metabolism, indicating that pathogenic or dysbiotic microbial overgrowth may represent one of the etiological factors underlying FBDs (81). Moreover, patients with FBDs frequently exhibit small intestinal bacterial overgrowth, and interventions specifically targeting this defined microbial abnormality have been shown to effectively alleviate related gastrointestinal symptoms, thereby underscoring the clinical value of precision-targeted, microbiota-based therapeutic strategies for FBDs (82, 83).

### 4.4 Limitations

This bibliometric and visualization-based assessment offers an extensive and systematic perspective on research trends and hotspots, yet it also carries several inherent limitations. First, the literature search was confined to the WoSCC and Scopus databases, which offer comprehensive coverage of high-quality publications and are considered ideal sources for bibliometric analysis (84, 85), but relevant studies indexed in other databases may still have been overlooked. Second, this study included only English-language publications, potentially excluding valuable studies published in other languages. Additionally, bibliometric analyses as a methodological constraint cannot serve as complete alternatives to systematic reviews, as they are unable to evaluate the quality and outcomes of individual studies. Finally, low citation counts typically indicate limited impact on the field, so we focus more on highly cited articles to analyze hotspots and trends, but citation metrics are inherently time-dependent, which may disadvantage more recent publications (86). While this study has limitations, its findings remain robust and credible, offering a comprehensive summary and a solid foundation for future investigations in this area.

## 5 Conclusion

This study systematically identifies the major research hotspots and emerging frontiers in GM research related to FBDs. The main findings include the following:

- Research exploring the role of GM in FBDs has attracted widespread global attention, with particularly strong contributions from researchers in China, the United States, Italy, the United Kingdom, and Australia. These countries represent the most prolific and influential contributors to this research field.
- In this research domain, Nutrients has the highest number of publications, whereas Gastroenterology demonstrates the highest citation frequency. Both Nutrients and Gastroenterology stand out as leading journals contributing significantly to advancements in this field.
- The current research focus centers on the mechanisms by which GM regulates FBDs via the gut-brain axis.
- Variations in the composition and metabolites of GM among different subtypes of FBDs represent a key research area.
- Intervention strategies for treating FBDs through the modulation of GM primarily emphasize specific dietary interventions, probiotics, prebiotics, and FMT.
- Clinical trials in this field have primarily focused on the regulatory role of core metabolites of the GM, such as SCFAs, in the symptoms of FBDs, while emphasizing integrated strategies for gut microecological modulation and exploring the impact of specific GM abnormalities in FBDs together with precision interventions.

In summary, this study offers an in-depth evaluation of the current research landscape concerning GM in FBDs, identifying key research hotspots and future trends. Through integrated bibliometric and visual analyses, the findings reveal the dominant research themes, collaboration networks, and evolving emphasis on mechanistic pathways, diagnostic biomarkers, and microbiota-based therapeutic strategies. These findings deepen our understanding of GM in FBDs. Collectively, this work provides a valuable reference for researchers seeking to explore novel directions and develop innovative strategies in future investigations.

## Data Availability

The original contributions presented in the study are included in the article/Supplementary material, further inquiries can be directed to the corresponding author.
