# Supplementary material for: Bibliometric and visual analysis of gut microbiota research in functional bowel disorders from 2016 to 2025
Source: Front Med (Lausanne). 2026 Jan 28;13:1735121. doi: 10.3389/fmed.2026.1735121 (PMC12891093; doi:10.3389/fmed.2026.1735121)
Supplement: Supplementary file 1 [file Table_1.DOCX]

| **Supplementary Material 1 The Scopus database and the PubMed database search formula** | |
| --- | --- |
| Scopus | TITLE-ABS-KEY((gut OR intestine OR bowel OR gastrointestine OR colon OR intestinal OR colorectal OR gastrointestinal OR enteric) AND (microbiome* OR microbiota* OR microbe* OR bacteria* OR microflora OR flora)) AND TITLE-ABS-KEY("functional bowel disorder*" OR "functional bowel disease*" OR "irritable bowel syndrome*" OR "irritable colon syndrome*" OR IBS OR "functional constipat*" OR "chronic idiopathic constipat*" OR "functional diarrh*" OR "functional abdominal bloat*" OR "functional abdominal distent*" OR "unspecified functional bowel disorder*" OR "opioid-induced constipat*") |
| PubMed | ((gut[Title] OR intestine[Title] OR bowel[Title] OR gastrointestine[Title] OR colon[Title] OR intestinal[Title] OR colorectal[Title] OR gastrointestinal[Title] OR enteric[Title]) AND (microbiome*[Title] OR microbiota*[Title] OR microbe*[Title] OR bacteria*[Title] OR microflora[Title] OR flora[Title])) AND (“functional bowel disorder*”[Title] OR “functional bowel disease*”[Title] OR “irritable bowel syndrome*”[Title] OR “irritable colon syndrome*”[Title] OR IBS[Title] OR “functional constipat*”[Title] OR “chronic idiopathic constipat*”[Title] OR “functional diarrh*”[Title] OR “functional abdominal bloat*”[Title] OR “functional abdominal distent*”[Title] OR “unspecified functional bowel disorder*”[Title] OR “opioid-induced constipat*”[Title]) AND ("2016/01/01"[Date - Publication] : "2025/09/14"[Date - Publication]) AND (English[Language]) Filters: Clinical Trial |
